# Potential noninvasive biomarkers for the malignant transformation of oral leukoplakia: A systematic review and meta‐analysis

**DOI:** 10.1002/cam4.6095

**Published:** 2023-05-18

**Authors:** Yan Huang, Qiufang Zhang, Zhenzhen Guo, Guanhong Deng, Ruibin Chen, Yanfen Zheng

**Affiliations:** ^1^ Department of Oral and Maxillofacial Surgery Stomatological Hospital of Xiamen Medical College Xiamen China; ^2^ Xiamen Key Laboratory of Stomotalogical Disease Diagnosis and Treatment Stomatological Hospital of Xiamen Medical College Xiamen China; ^3^ Department of Oral Mucosal Diseases Stomatological Hospital of Xiamen Medical College Xiamen China

**Keywords:** biomarkers, malignant transformation, noninvasive, oral cancer, oral leukoplakia

## Abstract

**Background:**

The rising cancer incidence in patients with oral leukoplakia (OL) highlights the importance of identifying potential biomarkers for high‐risk individuals and lesions because these biomarkers are useful in developing personalized management strategies for OL patients.

This study systematically searched and analyzed the literature on potential saliva and serum biomarkers for OL malignant transformation.

**Methods:**

PubMed and Scopus were searched for studies published up to April 2022. The primary outcome of this study was the difference in biomarker concentrations in saliva or serum samples from healthy control (HC), OL and oral cancer (OC) populations. Cohen's d with 95% credible interval was calculated and pooled using the inverse variance heterogeneity method.

**Results:**

A total of seven saliva biomarkers were analyzed in this paper, including interleukin‐1alpha, interleukin‐6 (IL‐6), interleukin‐6‐8, tumor necrosis factor alpha (TNF‐α), copper, zinc, and lactate dehydrogenase. IL‐6 and TNF‐α exhibited statistically significant deviations in comparisons between HC versus OL and OL versus OC. A total of 13 serum biomarkers were analyzed, including IL‐6, TNF‐α, C‐reactive protein, total cholesterol, triglycerides, high‐density lipoproteins, low‐density lipoproteins, albumin, protein, β2‐microglobulin, fucose, lipid‐bound sialic acid (LSA), and total sialic acid (TSA). LSA and TSA exhibited statistically significant deviations in comparisons between HC versus OL and OL versus OC.

**Conclusion:**

IL‐6 and TNF‐α in saliva have strong predictive values for OL deterioration, and LSA and TSA concentration levels in serum also have the potential to serve as biomarkers for OL deterioration.

## 1 INTRODUCTION

A white patch of oral mucosa is a condition defined as oral leukoplakia (OL) by the World Health Organization that cannot be erased or classified otherwise, either clinically or histopathologically.^1^ OL does not refer to the presence or absence of epithelial dysplasia at any stage; it is merely a clinical definition and is not related to a specific histology.^2^, ^3^ The prevalence of leukoplakia is estimated at 4.1% worldwide.^4^ OL can be clinically classified into homogeneous and nonheterogeneous lesions. Homogeneous leukoplakias are flat and uniform in appearance with small superficial cracks, and they are mostly benign and more common. Nonhomogeneous leukoplakias have a variety of features; they can be exophytic, nodular, white or red in color, flat and speckled, or papillary/verrucous.^2^, ^5^ Patients with OL are prone to developing oral cancer (OC).^6^ OC is closely associated with smoking, alcohol consumption, fungal, bacterial, and viral infections, and hormonal disorders. OL is one of the most common oral potentially malignant disorders (OPTMs, defined as any oral mucosal abnormality that is associated with a statistically increased risk of developing OC.^7^) and is responsible for approximately 11% of squamous carcinomas.^4^ Studies have shown a wide range of OL malignant transformation (MT), from 0.13% to 34.0%.^8^ The risk of MT is currently assessed by biopsy analysis of histopathological markers. The prognosis of OL may be affected by some clinical and pathological features, especially signs of epithelial dysplasia, such as asymmetric epithelial stratification, myoepithelial basocellular hyperplasia, dyskeratosis, hyperchromatic nuclei, and pleomorphism.^9^ However, studies have shown that grading epithelial dysplasia is difficult in the clinical setting, and treatment planning of OL patients is usually made on the basis of subjective and poorly reproducible judgment. Patients diagnosed with OPMDs can develop cancer anywhere in the mouth during their lifetime. The cancer does not necessarily occur at the site of the visibly altered mucosa. A key challenge for clinicians managing patients with OPMD is identifying the small proportion of patients who are most likely to develop future malignancy.^7^ Therefore, there is growing interest in minimally invasive biomarkers for diagnostic procedures. Oral carcinogenesis usually stems from a genetic mutation due to long‐term exposure to carcinogens that change tissue homeostasis and increase the possibility of MT. These changes occur in multiple pathways, such as cell proliferation signaling, evasion of immune destruction, invasion and angiogenesis, and reprogramming of cellular energetics.^10^ The proteins and genes involved in these pathways are frequently deregulated during oral carcinogenesis.^11^, ^12^, ^13^ Saliva sample analysis can be noninvasive, rapidly collected, and easily processed. These advantages make saliva analysis more attractive than biopsy‐based histopathological analysis or the use of another matrix and is a promising option for the diagnosis and prognosis of OC.

This study systematically searched and analyzed the literature on potential salivary and serum biomarkers for OL MT. We hope that our study will provide strong clues for finding biomarkers, especially noninvasive biomarkers, for OL MT.

## 2 MATERIALS AND METHODS

The study was designed according to the Preferred Reporting Items for Systematic Reviews and Meta‐Analyses (PRISMA) requirements.^14^ This systematic review and meta‐analysis were registered in the INPLASY database under the registration number INPLASY202250166 (https://inplasy.com/inplasy‐2022‐5‐0166/).

### 2.1 Literature search strategy

PubMed and Scopus were searched for studies published up to April 2022. The literature search strategy was (“oral” AND “leukoplakia”) AND (“saliva” OR “spit” OR “serum”). English was set as a language restriction. We also checked the reference list of all identified articles for additional relevant studies, including hand‐searching reviews and previous meta‐analyses.

### 2.2 Eligibility criteria and selection of studies

The inclusion criteria for selecting the articles were as follows: The case–control studies reported research on biomarkers in human saliva or serum samples. The samples were obtained from individuals with OL and proliferative verrucous leukoplakia. The articles reported concentrations of biomarkers in OL patients as well as samples from OC patients and healthy controls (HCs) and their association with possible malignant degeneration. Two authors independently examined the titles and abstracts of citations. Full texts of potentially eligible studies were obtained, and disagreements were resolved by discussion.

### 2.3 Data extraction

Two reviewers extracted the data independently. The first author, publication year, study design, number of patients in each group, assay method, country, biomarker, and concentration of biomarkers in each arm were recorded. We tabulated the concentrations of the biomarkers of each study in different groups and sorted out the data of the planned comparison group for the synthesis. If the concentration units of the biomarker were inconsistent, they were converted to the same unit.

### 2.4 Quality assessment

The Newcastle–Ottawa Scale (NOS) was used to evaluate the quality of the included studies. It evaluates case–control studies through three categories and eight items, including study population selection, comparability, exposure evaluation and outcome evaluation. The NOS adopts the semiquantitative principle of the star system in the evaluation of study quality, with a full score of nine stars.

### 2.5 Statistical analysis

This study analyzed the standard mean difference of the saliva or serum concentration of the biomarkers in HC versus OL and OL versus OC populations. The synthesis results of all biomarkers extracted from the included studies were tabulated and the results of each study and synthesis were visualized using forest plots. Only biomarkers with a significant difference in both comparisons could be considered potential biomarkers for predicting OL MT. Cohen's d with a 95% credible interval (CI) was calculated and pooled using the inverse heterogeneity (IVhet) of variances method. IVhet is an improved alternative to the random effects (RE) model for meta‐analysis of heterogeneous studies. It is shown that the known issues of underestimation of the statistical error and spuriously overconfident estimates with the RE model can be resolved by the use of an estimator under the fixed effect model assumption with a quasi‐likelihood based variance structure. Extensive simulations confirm that this estimator retains a correct coverage probability and a lower observed variance than the RE model estimator, regardless of heterogeneity.^15^ The Chi^2^ test and Higgins I^2^ statistics were used to assess heterogeneity among the included studies.^16^ In addition, sensitivity analyses were performed by leave‐one‐out analysis. Publication bias was not assessed because fewer than 10 studies were included in each comparison. The meta‐analysis was conducted in MetaXL 5.3 (EpiGear International).

## 3 RESULTS

### 3.1 Literature search and characteristics of the included studies

**FIGURE 1:**
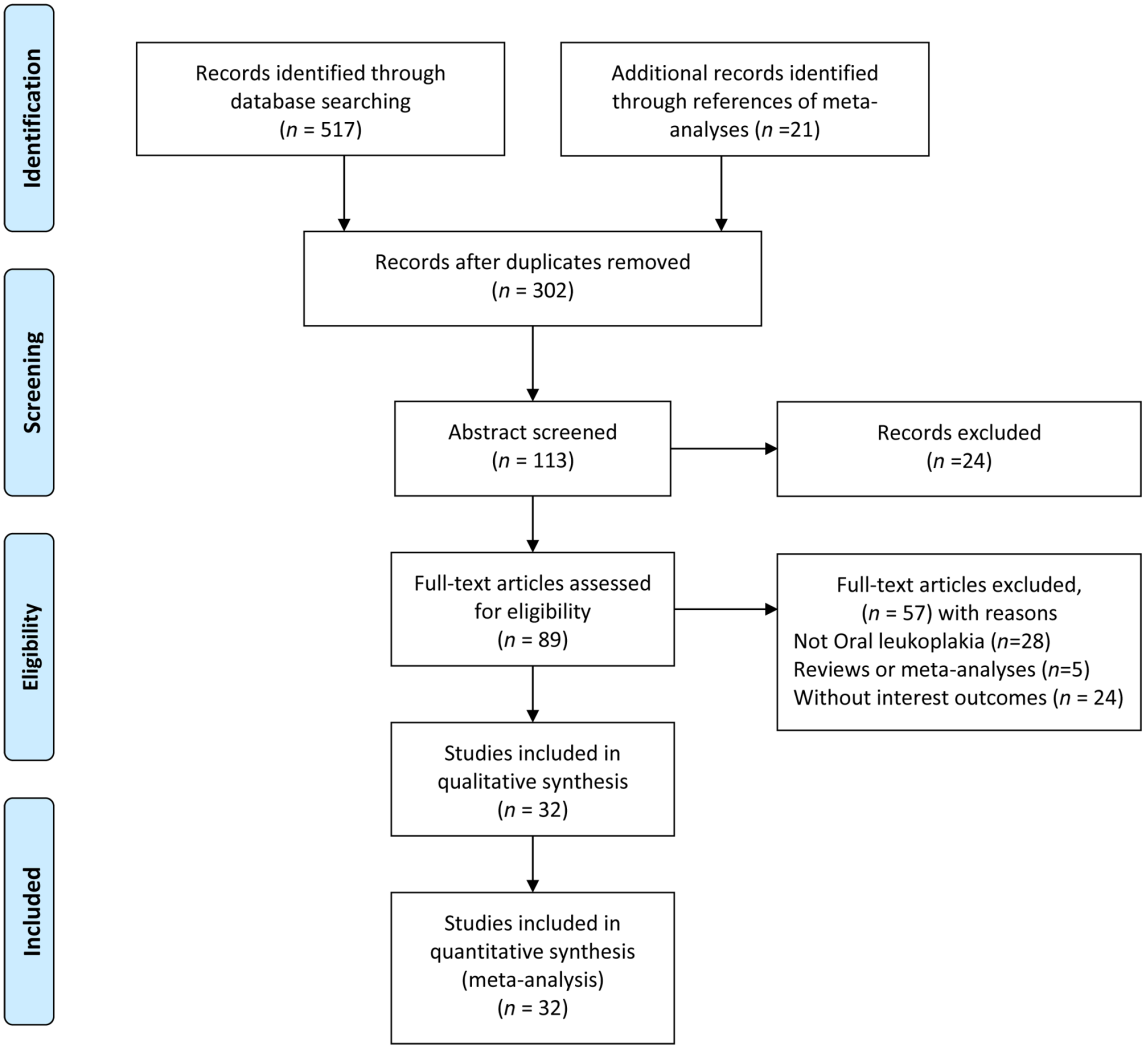
Flowchart of study selection.

A total of 538 studies were obtained through preliminary retrieval, of which 113 were subjected to full‐text evaluation. Finally, 32 articles involving 3223 subjects (including 1050 OL patients, 957 OC patients, and 1216 HCs) were included in this meta‐analysis (Figure 1). The characteristics of the included studies are shown in Table 1. All included studies were case–control studies, of which 10 studies investigated salivary biomarkers, 17 focused on serum biomarkers, and six analyzed both. The studies covered six groups of biomarkers, including cytokines (interleukin‐1 alpha [IL‐1α], IL‐6, IL‐8, tumor necrosis factor‐alpha [TNF‐α], and C‐reactive protein [CRP]), lipid profiles (total cholesterol [TC], triglycerides [TG], high‐density lipoprotein [HDL], and low‐density lipoprotein [LDL]), proteins (albumin, protein, and β2‐microglobulin [β2‐M]), glycoconjugates (fucose, lipid‐bound sialic acid (LSA) and total sialic acid (TSA)), trace elements ((copper and zinc), and cytoplasmic enzymes lactate dehydrogenase (LDH)). The NOS scale was used to evaluate the studies' quality, and the scores are shown in Table 1. Due to the small sample sizes, the studies were considered to have average quality, and most studies lacked a detailed description of whether important additional factors were controlled for.

**TABLE 1:** Characteristics of included studies.

First author	Publication year	Biomarkers	Sample size	OL group	OC group	HC group	Assays	Serum /Salivary	Country	Selection	Comparability	Exposure
Anil^17^	1995	β2‐M	90	30	30	30	β2‐micro radioimmunoassay kit	Serum	India	☆☆☆	☆	☆☆
Babiuch^18^	2020	IL‐1α, IL‐6, IL‐8, TNF‐α	42	21	14	7	ELISA	Salivary	Poland	☆☆	☆	☆☆
Bagan^19^	2016	IL‐6	60	20	20	20	ELISA	Both	Spain	☆☆☆	☆	☆☆
Bose^20^	2013	Fucose, TSA	133	27	26	80	Dische and Shettles method	Serum	India	☆☆☆	☆	☆☆
Brailo^21^	2006	IL‐6, TNF‐α	64	30		34	ELISA	Salivary	Croatia	☆☆☆	☆☆	☆☆
Brailo^22^	2012	IL‐6, TNF‐α	88	29	28	31	ELISA	Both	Croatia	☆☆☆	☆☆	☆☆
Chang^23^	2013	CRP, IL‐6	308	46	151	111	ELISA	Serum	China	☆☆☆	☆	☆☆
Chen^24^	2011	CRP	77	59	18		Detected with a high‐sensitivity assay using an auto‐analyzer	Serum	Taiwan	☆☆☆	☆	☆☆
Dikova^25^	2021	IL‐1α, IL‐6, IL‐8, TNF‐α	157	66	66	25	Sensitive bead‐based multiplex immunoassay	Salivary	Spain	☆☆☆	☆☆	☆☆
G^26^	2019	TNF‐α	90	30	30	30	ELISA	Salivary	India	☆☆☆	☆☆	☆☆
Ganavi^27^	2014	HDL, LDL, TC, Tg	30	10	10	10	ELISA	Serum	India	☆☆	☆	☆☆
Joshi^28^	2014	LDH	90	30	30	30	Agarose gel electrophoresis method	Salivary	India	☆☆☆	☆	☆☆
Kaur^29^	2015	IL‐6, IL‐8, TNF‐α	100	50		50	ELISA	Both	Belgium	☆☆☆	☆☆	☆☆
Kumar^30^	2012	HDL, LDL, TC, Tg	90	30	30	30	Based on the principle of photometry	Serum	India	☆☆☆	☆	☆☆
Kumar^31^	2019	Fucose	75	25	25	25	The method of Dische and Shettles	Serum	India	☆☆☆	☆	☆☆
MEISEL^32^	2010	CRP, HDL, LDL, TC	369	123		246	Standard laboratory methods CRP: immuno‐nephelometry	Serum	Germany	☆☆☆	☆	☆☆
Metgud^33^	2014a	Albumin	45	15	15	15	Bromocresol green method	both	India	☆☆	☆	☆☆
More^34^	2017	Albumin, protein	150	50	50	50	EBRA EM 200 semi‐quantitive analyzer	Serum	India	☆☆☆	☆	☆☆
Nayyar^35^	2012	Albumin, protein	65	10	30	25	Biuret method	Serum	India	☆☆☆	☆	☆☆
Panda^36^	2020	LDH	80	40		40	Enzymatic UV‐Kinetic method	Both	India	☆☆☆	☆	☆☆
Panneer Selvam^37^	2015	IL‐6	75	25	25	25	ELISA	Salivary	India	☆☆☆	☆	☆☆
Patel^38^	2015	LDH	75	25	25	25	Biovision Lactate Dehydrogenase Activity Colorimetric Assay Kit	Salivary	India	☆☆☆	☆	☆☆
Pereira^39^	2015	LDH	80	30	30	20	Processed on Express 550 Ceiba Corning auto analyzer for spectrometry	Serum	India	☆☆☆	☆	☆☆
Rai^40^	2015	Fucose	60	20	20	20	Fucose estimation was done According to the method of Winzler	Serum	Malaysia	☆☆☆	☆	☆☆
Rajpura^41^	2005	LSA, TSA	69	8	41	20	Serum TSA contents of serum were determined using a periodiate–thiobarbituric acid method	Serum	India	☆☆☆	☆	☆☆
Saddiwal^42^	2017	β2‐M	45	15	15	15	Chemi‐luminescent immunometric assay	Serum	India	☆☆	☆	☆☆
Sawhney^43^	2011	LSA, TSA	75	25	25	25	TSA: periodatethiobarbituric acidmethod LSA: resorcinol reagent	Serum	India	☆☆☆	☆	☆☆
Scully^44^	1981	β2‐M	63	10	26	27	Duplicate by radioimmunoassay	Serum	Scotland	☆☆☆	☆	☆☆
Shetty^45^	2015	LDH	150	50	50	50	Cu, Zn: GBC Avanta atom Absorption spectrophotometer; Fe: Bathophenanthroline method	Salivary	India	☆☆☆	☆	☆☆
Shetty^46^	2012	Copper, Zinc	75	25	25	25	Using the standard kit and measured sphectrophotometrically at 340 nm	Salivary	India	☆☆☆	☆	☆☆
Singh^47^	2021	HDL, TC, Tg	103	26	52	25	Enzymatic CHOD PAP method	Both	India	☆☆☆	☆	☆☆
Varghese^48^	1987	Copper, Zinc	150	50	50	50	atomic absorption Spectrophotometryusing a Perkin‐Elmer400A atomic absorption spectrophotometer.	Salivary	India	☆☆☆	☆	☆☆
Total studies 32			3223	1050	957	1216						

Abbreviations: β2‐M, β2‐microglobulin; CRP, C‐reactive protein; HC, health control; HCI, high confidence interval; HDL, high density lipoproteins; IL‐1α, interleukin‐1alpha; IL‐6, interleukin‐6; LCI, low confidence interval; LDH, lactate dehydrogenase; LDL, low density lipoproteins; LSA, lipid bound sialic acid; OC, oral cancer; OL, Oral leukoplakia; TC, total cholesterol; TG, triglycerides; TNF‐α, tumor necrosis factor alpha; TSA, total sialic acid.

### 3.2 Data synthesis

#### 3.2.1 Salivary Biomarkers for the MT of OL

A total of seven salivary biomarkers were analyzed in this paper, including IL‐1α, IL‐6, IL‐8, TNF‐α, copper, zinc, and LDH. IL‐6 and TNF‐α exhibited statistically significant deviations in comparisons between HC versus OL and OL versus OC. The standardized mean difference Cohen's d values for the IL‐6 concentration level in the saliva of HC versus OL and OL versus OC were − 1.07 [95% CI‐1.86, ‐0.28] and − 1.01 [95% CI‐1.80, ‐0.22], respectively. The standardized mean difference Cohen's d values for the TNF‐α concentration level in the saliva of HC versus OL and OL versus OC were − 0.83 [95% CI ‐1.61, ‐0.05] and − 0.86 [95% CI ‐1.58, ‐0.13], respectively (Table 2, Figures S1–S4). The results suggested that the concentration levels of IL‐6 and TNF‐α in the saliva of HCs and OL and OC patients exhibited an increasing trend, while no significant changes were observed in the other 5 salivary biomarkers, including IL‐1α, IL‐8, copper, zinc, and LDH (Figures S5–S14).

**TABLE 2:** Outcomes.

Type	Biomarkers	Serum/saliva	OL versus HC					OL versus OC				
			Cohen's d	LCI 95%	HCI 95%	I^2^	Number of studies included	SMD	LCI 95%	HCI 95%	I^2^	Number of studies included
Cytokines	IL‐1α	Saliva	−0.30	−0.71	0.10	0.00	2	−0.10	−3.45	3.24	96.78	2
	***IL‐6***	***Saliva***	***−1.01***	***−1.80***	***−0.22***	***91.59***	***7***	***−1.07***	***−1.86***	***−0.28***	***84.69***	***5***
	IL‐6	Serum	−0.74	−2.51	1.02	96.98	4	−0.17	−0.49	0.15	26.09	3
	IL‐8	Saliva	***−0.66***	***−1.07***	***−0.24***	***0.00***	***2***	−0.95	−7.62	5.73	97.46	2
	***TNF‐α***	***Saliva***	***−0.86***	***−1.58***	***−0.13***	***89.85***	***6***	***−0.83***	***−1.61***	***−0.05***	***85.71***	***4***
	TNF‐α	Serum	−0.79	−2.72	1.14	96.83	2	‐	‐	‐	‐	‐
	CRP	Serum	−0.21	−0.71	0.30	81.45	2	−0.20	−0.48	0.08	0.00	2
Lipid profile	TC	Serum	−0.29	−0.94	0.35	75.69	4	0.41	−0.21	1.04	66.35	3
	TG	Serum	−0.25	−0.64	0.15	19.40	3	0.77	−1.71	3.24	96.36	3
	HDL	Serum	0.36	−0.89	1.62	91.08	4	0.53	−2.59	3.65	97.31	3
	LDL	Serum	−0.24	−0.85	0.37	68.17	3	2.22	−4.45	8.90	98.42	2
Proteins	Albumin	Serum	***0.72***	***0.39***	***1.06***	***92.50***	***3***	0.16	−0.16	0.48	87.37	3
	Protein	Serum	0.26	−1.08	1.60	87.53	2	−0.29	−1.57	0.99	87.26	2
	β2‐M	Serum	***−0.85***	***−1.36***	***−0.34***	***38.88***	***3***	−1.07	−2.16	0.02	83.66	3
Glycoconjugates	Fucose	Serum	−0.21	−0.71	0.30	81.45	2	−1.41	−3.23	0.42	94.38	3
	***LSA***	***Serum***	***−2.54***	***−4.05***	***−1.03***	***82.16***	***2***	***−2.54***	***−3.39***	***−1.69***	***51.13***	***2***
	***TSA***	***Serum***	***−2.25***	***−3.35***	***−1.16***	***77.46***	***3***	***−2.01***	***−3.89***	***−0.13***	***93.19***	***3***
Trace elements	Copper	Saliva	−0.06	−0.65	0.54	78.30	2	0.38	−0.58	1.35	91.25	2
	Zinc	Saliva	0.97	−1.29	3.24	97.82	2	1.51	−2.20	5.21	98.57	2
Cytoplasmic enzyme	LDH	Saliva	−4.44	−12.06	3.18	98.27	4	***−2.85***	***−3.96***	***−1.74***	***82.40***	***3***

*Note*: Bold indicates that the mean difference of the concentration of this biomarker was significantly different in patients with OL versus HC, and also significantly different in patients with OL vs OC.

Abbreviations: β2‐M, β2‐microglobulin; CRP, C‐reactive protein; HC, health control; HCI, high confidence interval; HDL, high density lipoproteins; IL‐1α, interleukin‐1alpha; IL‐6, interleukin‐6; LCI, low confidence interval; LDH, lactate dehydrogenase; LDL, low density lipoproteins; LSA, lipid bound sialic acid; OC, oral cancer; OL, Oral leukoplakia; TC, total cholesterol; TG, triglycerides; TNF‐α, tumor necrosis factor alpha; TSA, total sialic acid.

#### 3.2.2 Serum Biomarkers for the MT of OL

A total of 13 serum biomarkers were analyzed, including IL‐6, TNF‐α, CRP, TC, TG, HDL, LDL, albumin, protein, β2‐M, fucose, LSA, and TSA. LSA and TSA exhibited statistically significant deviations in comparisons between HC versus OL and OL versus OC. The standardized mean difference Cohen's d values for the LSA concentration level were − 2.54 [95% CI‐3.39, ‐1.69] in the serum of HC versus OL and − 2.54 [95% CI‐4.05, ‐1.03] in OL versus OC. For the TSA concentration level, the standardized mean difference Cohen's d between HC and OL serum was−2.01 [95%CI‐3.89, ‐0.13], and that between OL and OC serum was −2.25 [95% CI ‐3.35, ‐1.16] (Table 2, Figures S15–S18). These results showed that the concentration levels of LSA and TSA in the serum of HC, OL, and OC indicated an increasing trend, while the other 11 serum biomarkers (IL‐6, TNF‐α, CRP, TC, TG, HDL, LDL, albumin, protein, β2‐M, and fucose) displayed no differences (Figures S19–S39).

### 3.3 Sensitivity analyses

Only IL‐6 remained stable as we tested the sensitivity of the potential salivary biomarkers IL‐6 and TNF‐α, as well as the serum biomarkers LSA and TSA. The statistical significance of the other three potential biomarkers varied with the removal of factors from the analysis one by one. See Table 3 for details.

**TABLE 3:** Sensitivity analyses.

Biomarker	Comparison	Excluded study	Pooled d	LCI 95%	HCI 95%	I^2^
Salivary IL‐6	OL versus. HC	Babiuch 2020	−1.03	−1.89	−0.16	92.92
		Bagan 2016	−0.98	−1.89	−0.07	92.94
		Brailo 2006	−1.03	−2.00	−0.07	92.96
		Brailo 2012	−1.21	−2.07	−0.36	91.01
		Dikova 2021	−1.11	−2.06	−0.17	92.63
		Kaur 2015	−0.67	−1.00	−0.35	44.98
		Panneer Selvam 2015	−1.03	−1.97	−0.09	92.97
	OL versus OC	Babiuch 2020	−0.96	−1.69	−0.23	82.26
		Bagan 2016	−0.96	−1.76	−0.15	84.04
		Brailo 2012	−1.23	−2.21	−0.26	84.51
		Dikova 2021	−1.34	−2.30	−0.38	85.47
		Panneer Selvam 2015	−0.98	−1.94	−0.01	86.88
Salivary TNF‐α	HC versus OL	Babiuch 2020	−0.86	−1.66	−0.06	91.88
		Brailo 2006	−0.92	−1.82	−0.03	91.62
		Brailo 2012	−1.15	−1.69	−0.60	77.99
		***Dikova 2021***	***−0.88***	***−1.79***	***0.04***	***91.86***
		***G 2019***	***−0.74***	***−1.56***	***0.08***	***90.70***
		***Kaur 2015***	***−0.58***	***−1.27***	***0.10***	***85.46***
	OL versus OC	Babiuch 2020	−0.70	−1.27	−0.13	75.80
		Brailo 2012	−1.07	−1.85	−0.28	79.19
		***Dikova 2021***	***−0.72***	***−1.92***	***0.48***	90.05
		***G 2019***	***−0.84***	***−2.01***	***0.34***	90.47
Serum TSA	HC versus OL	Bose 2013	−3.03	−3.70	−2.36	0.00
		Rajpura 2005	−2.11	−3.39	−0.83	83.87
		Sawhney 2011	−1.97	−3.59	−0.36	79.34
	OL versus OC	Bose 2013	−1.42	−2.00	−0.84	24.35
		***Rajpura 2005***	***−2.47***	***−5.49***	***0.55***	95.27
		***Sawhney 2011***	***−2.34***	***−5.82***	***1.14***	96.32

*Note*: Bold Italic indicates this study is sensitive.

Abbreviations: HC, health control; HCI, high confidence interval; IL‐6, interleukin‐6; LCI, low confidence interval; OC, oral cancer; OL, Oral leukoplakia; TNF‐α, tumor necrosis factor alpha; TSA, total sialic acid.

## 4 DISCUSSION

A total of 32 studies involving 3223 participants were included in this study, and six kinds of potential biomarkers in saliva and serum were analyzed, including cytokines, lipid profiles, proteins, glycoconjugates, cytoplasmic enzymes, and trace elements. The preponderance of evidence indicates that certain molecular alterations can be considered biomarkers for the risk of MT. This study aims to find statistically consistent evidence that these molecules may serve as potential biomarkers for OL deterioration.

The present study revealed that in OL and OC patients, salivary cytokines, such as IL‐6 and TNF‐α, showed statistically consistent deviations, suggesting that OL patients had higher IL‐6 and TNF‐α concentration levels in saliva than healthy people but had lower IL‐6 and TNF‐α concentrations than OC patients. This finding suggests that altered cytokine responsiveness is not only closely associated with OC development but also correlated with premalignant lesions such as OL; this result was consistent with that in previous studies.^18^, ^49^ In normal cells, growth inhibition is accompanied by stimulation with proinflammatory cytokines, and in OC cells, upregulation of positive cell cycle regulators such as nuclear factor kappa B (NF‐κB) occurs with the stimulation of proinflammatory cytokines. Various studies have shown that abnormal activation of NF‐κB is associated with the development and progression of human cancers, including head and neck squamous cell carcinoma. This leads to the upregulation of antiapoptotic factors, proangiogenic factors and proinflammatory cytokines responsible for tumor growth.^50^ When a heterodimeric receptor, which is composed of the common cytokine receptor signal‐transducing subunit gp130 ^51^ and ligand‐binding IL‐6α chain, binds to IL‐6 as a multifunctional cytokine, the JAK family of tyrosine kinases is activated, and multiple pathways are stimulated, involving STATs, PI3Ks, MAPKs, and other signaling proteins.^52^ Various subcomponents of these pathways are involved in the oncogenic cascade, affecting various cancers, including oral squamous cell carcinoma (OSCC) and premalignant oral lesions.^53^, ^54^, ^55^

TNF‐α is also a pleiotropic cytokine. The TNF‐TNF receptor system is known as an important player in the process of MT that undergoes programmed cell death, angiogenesis, proliferation, and inflammation.^56^ TNF‐α may directly induce DNA damage in cells, which is related to cell MT, by inducing reactive oxygen species.^57^ Moreover, TNF family members are also involved in immunosuppression.^58^ According to G et al,^26^ the specificity and sensitivity of salivary TNF‐α were 96.7% and 100%, respectively, in OSCC and 93.3% and 90% in oral epithelial dysplasia. The significantly reliable and valid values suggest that salivary TNF‐α can be used as a biomarker for OSCC and oral epithelial dysplasia. Saliva can reflect the healthy state and systemic disease state of individuals. Thus, salivary biomarkers are ideal for making pathological diagnoses. Because saliva directly comes from the oral cavity, saliva testing is an ideal choice for assessing potential malignancy and malignant oral lesions and identifying individuals at risk.^59^, ^60^ Therefore, we believe that IL‐6 and TNF‐α are potential biomarkers for OL deterioration and can be used as useful screening tools.

The levels of IL‐6 and TNF‐α in saliva vary with the progression of OL; therefore, they can be used for the diagnosis and prognosis of OL MT, providing clinicians with a valuable noninvasive procedure as a clinical finding supplement.

According to our study, the molecular concentration levels of LSA and TSA in serum glycoconjugates also show statistically consistent deviations in OL and OC. The surface of the cell membrane is mainly composed of glycolipids and glycoproteins. Sialic acid, a family of acetylated derivatives of neuraminic acid, is widely found in mammals and usually occurs as a terminal component at the nonreducing end of carbohydrate chains of glycolipids and glycoproteins.^61^ Sialic acid, mainly LSA, is an important factor in determining cell surface properties and is associated with immunogenicity, adhesion, and cell invasiveness.^62^ Any alteration in the intracellular microenvironment may result in a change in the composition of the surface membrane. The altered carbohydrate composition of glycolipids and glycoproteins on the surface of malignant cells is responsible for antigenicity, cell adhesion, aberrant intercellular recognition, and invasiveness. These glycolipids and glycoproteins can be released into the serum by shedding, secretion, and increased turnover.^63^ Therefore, LSA and TSA concentration levels in serum have potential diagnostic and prognostic values for OL deterioration.

Most biomarkers showed no statistically consistent deviations in our study. LDH concentration levels in saliva showed statistically significant deviations in OL versus OC but not in HC versus OL. The enzyme LDH is found in cells of almost all body tissues. Increased LDH levels are due to an increased mitotic index and lactic acid production by tumor cells due to the breakdown of glycoproteins. Our results, however, cannot deny the potential role of salivary LDH.

Several studies have found significant hypocholesterolemia and varied serum lipoprotein (TC, TG, HDL, LDL) levels in OC and oral precancerous lesions.^64^, ^65^, ^66^ The concentration levels of copper and zinc tend to decrease in the blood of patients with head and neck cancer.^67^, ^68^ However, no difference was detected in our meta‐analysis in either the OL versus HC comparison or the OL versus OC comparison.

The oxidation of proteins plays an important role in the pathogenesis of OC.^35^, ^69^ Hyperproteinemia is commonly observed in oral malignancies and is expressed as cachexia.^70^ However, no deviation in serum albumin, protein, or β2‐M was detected in our pooled values for OL versus OC.

All studies included in this meta‐analysis were case–control studies of average quality and small sample sizes from hospitals rather than communities. The assay method and other methods and means used varied from study to study, resulting in considerable heterogeneity in the biomarker concentration levels. Our findings need to be interpreted with caution, although we have reduced the impact of the assay method by applying Cohen's d rather than the weighted mean difference to evaluate the difference in concentrations. Furthermore, due to the large number of biomarkers and the scattered focus of research, the studies included for each biomarker are insufficient. The sensitivity analysis confirmed the potential of salivary IL‐6 as a biomarker, but also suggested the unstable results of salivary TNF‐α and serum TSA. Since only two studies were involved in the synthesis of serum LSA, the sensitivity analysis was not performed. Its results should also be interpreted with caution. Due to the large number of salivary biomarkers, we mainly considered three aspects when selecting the target biomarkers in this study. First, we reviewed previous reviews and meta‐analyses for the hot OL biomarkers to make a preliminary selection.^71^, ^72^, ^73^, ^74^, ^75^ Second, since the same disease was studied with different instruments and experimental designs, it is not surprising that different biomarkers were proposed for the same pathology, ELISA was chosen for our study.^7^ Third, understudied biomarkers were abandoned by full‐text reading when the search strategy was chosen for inclusion in the study. Due to the subjective factors in the above process, OL salivary and serum biomarkers involved in this study may still be lacking. We found only one biomarker with stable statistical difference, providing clues for future large‐sample, multi‐instrument verification experiments. It is expected that follow‐up research will focus on a certain number of potential biomarkers, such as salivary IL‐6 synthesized in this study, and find more accurate evidence.

## 5 CONCLUSION

Our study shows that IL‐6 and TNF‐α in saliva have strong predictive values for OL deterioration, and LSA and TSA concentration levels in serum also have the potential to serve as biomarkers for OL deterioration. This study is obviously insufficient in quantity and quality despite the statistically consistent evidence. Some larger and multicenter studies are needed to ensure the reliability of the consistent evidence before these biomarkers are applied in clinical practice.

## AUTHOR CONTRIBUTIONS

**Yan Huang:** Writing – original draft. **Qiufang Zhang:** Formal analysis. **Zhenzhen Guo:** Data curation; validation. **Guanhong Deng:** Data curation; validation. **Ruibin Chen:** Formal analysis. **Yanfen Zheng:** Writing – review and editing.

## FUNDING INFORMATION

This work was supported by the Introduced talents' research fund of Stomatological Hospital of Xiamen Medical College [grant number RCYJ004]; the Xiamen Medical College First Class Program [grant number XBJK2022009]; and the Xiamen Medical College educational Program [grant number XBJG2021007].

## CONFLICT OF INTEREST STATEMENT

The authors declare that there are no conflicts of interest.

## ETHICS STATEMENT

Not applicable.

## PATIENT CONSENT

Not applicable.

## PERMISSION TO REPRODUCE MATERIAL FROM OTHER SOURCES

Not applicable.

## CLINICAL TRIAL REGISTRATION

Not applicable.

## Supporting information


**Figure S1.** Forest plot of standard mean difference Cohen’s *d* of salivary IL‐6 concentration in patients with HC versus patients with OL. IL‐6, interleukin‐6; HC, health control; OL, Oral leukoplakia; CI, credible interval
**Figure S2**. Forest plot of standard mean difference Cohen’s *d* of salivary IL‐6 concentration in patients with OL versus patients with OC. IL‐6, interleukin‐6; OL, Oral leukoplakia; OC, oral cancer; CI, credible interval
**Figure S3**. Forest plot of standard mean difference Cohen’s *d* of salivary TNF‐α concentration in patients with HC versus patients with OL. TNF‐α, tumor necrosis factor alpha; HC, health control; OL, Oral leukoplakia; CI, credible interval
**Figure S4**. Forest plot of standard mean difference Cohen’s *d* of salivary TNF‐α concentration in patients with OL versus patients with OC. TNF‐α, tumor necrosis factor alpha; OL, Oral leukoplakia; OC, oral cancer; CI, credible interval
**Figure S5**. Forest plot of standard mean difference Cohen’s *d* of salivary IL‐1α concentration in patients with HC versus patients with OL. IL‐1α, interleukin‐1alpha; HC, health control; OL, Oral leukoplakia; CI, credible interval
**Figure S6**. Forest plot of standard mean difference Cohen’s *d* of salivary IL‐1α concentration in patients with OL versus patients with OC. IL‐1α, interleukin‐1alpha; OL, Oral leukoplakia; OC, oral cancer; CI, credible interval
**Figure S7**. Forest plot of standard mean difference Cohen’s *d* of salivary IL‐8 concentration in patients with HC versus patients with OL. IL‐8, interleukin‐8; HC, health control; OL, Oral leukoplakia; CI, credible interval
**Figure S8**. Forest plot of standard mean difference Cohen’s *d* of salivary IL‐8 concentration in patients with OL versus patients with OC. IL‐8, interleukin‐8; OL, Oral leukoplakia; OC, oral cancer; CI, credible interval
**Figure S9**. Forest plot of standard mean difference Cohen’s *d* of salivary Copper concentration in patients with HC versus patients with OL. HC, health control; OL, Oral leukoplakia; CI, credible interval
**Figure S10**. Forest plot of standard mean difference Cohen’s *d* of salivary Copper concentration in patients with OL versus patients with OC. OL, Oral leukoplakia; OC, oral cancer; CI, credible interval
**Figure S11**. Forest plot of standard mean difference Cohen’s *d* of salivary Zinc concentration in patients with HC versus patients with OL. HC, health control; OL, Oral leukoplakia; CI, credible interval
**Figure S12**. Forest plot of standard mean difference Cohen’s *d* of salivary Zinc concentration in patients with OL versus patients with OC. OL, Oral leukoplakia; OC, oral cancer; CI, credible interval
**Figure S13**. Forest plot of standard mean difference Cohen’s *d* of salivary LDH concentration in patients with HC versus patients with OL. LDH, lactate dehydrogenase; HC, health control; OL, Oral leukoplakia; CI, credible interval
**Figure S14**. Forest plot of standard mean difference Cohen’s *d* of salivary LDH concentration in patients with OL versus patients with OC. LDH, lactate dehydrogenase; OL, Oral leukoplakia; OC, oral cancer; CI, credible interval
**Figure S15**. Forest plot of standard mean difference Cohen’s *d* of serum LSA concentration in patients with HC versus patients with OL. LSA, lipid bound sialic acid; HC, health control; OL, Oral leukoplakia; CI, credible interval
**Figure S16**. Forest plot of standard mean difference Cohen’s *d* of serum LSA concentration in patients with OL versus patients with OC. LSA, lipid bound sialic acid; OL, Oral leukoplakia; OC, oral cancer; CI, credible interval
**Figure S17**. Forest plot of standard mean difference Cohen’s *d* of serum TSA concentration in patients with HC versus patients with OL. TSA, total sialic acid; HC, health control; OL, Oral leukoplakia; CI, credible interval
**Figure S18**. Forest plot of standard mean difference Cohen’s *d* of serum TSA concentration in patients with OL versus patients with OC. TSA, total sialic acid; OL, Oral leukoplakia; OC, oral cancer; CI, credible interval
**Figure S19**. Forest plot of standard mean difference Cohen’s *d* of serum IL‐6 concentration in patients with HC versus patients with OL. IL‐6, interleukin‐6; HC, health control; OL, Oral leukoplakia; CI, credible interval
**Figure S20**. Forest plot of standard mean difference Cohen’s *d* of serum IL‐6 concentration in patients with OL versus patients with OC. IL‐6, interleukin‐6; OL, Oral leukoplakia; OC, oral cancer; CI, credible interval
**Figure S21**. Forest plot of standard mean difference Cohen’s *d* of serum TNF‐α concentration in patients with HC versus patients with OL. TNF‐α, tumor necrosis factor alpha; HC, health control; OL, Oral leukoplakia; CI, credible interval
**Figure S22**. Forest plot of standard mean difference Cohen’s *d* of serum CRP concentration in patients with HC versus patients with OL. CRP, C‐reactive protein; HC, health control; OL, Oral leukoplakia; CI, credible interval
**Figure S23**. Forest plot of standard mean difference Cohen’s *d* of serum CRP concentration in patients with OL versus patients with OC. CRP, C‐reactive protein; OL, Oral leukoplakia; OC, oral cancer; CI, credible interval
**Figure S24**. Forest plot of standard mean difference Cohen’s *d* of serum TC concentration in patients with HC versus patients with OL. TC, total cholesterol; HC, health control; OL, Oral leukoplakia; CI, credible interval
**Figure S25**. Forest plot of standard mean difference Cohen’s *d* of serum TC concentration in patients with OL versus patients with OC. TC, total cholesterol; OL, Oral leukoplakia; OC, oral cancer; CI, credible interval
**Figure S26**. Forest plot of standard mean difference Cohen’s *d* of serum TG concentration in patients with HC versus patients with OL. TG, triglycerides; HC, health control; OL, Oral leukoplakia; CI, credible interval
**Figure S27**. Forest plot of standard mean difference Cohen’s *d* of serum TG concentration in patients with OL versus patients with OC. TG, triglycerides; OL, Oral leukoplakia; OC, oral cancer; CI, credible interval
**Figure S28**. Forest plot of standard mean difference Cohen’s *d* of serum HDL concentration in patients with HC versus patients with OL. HDL, high density lipoproteins HC, health control; OL, Oral leukoplakia; CI, credible interval
**Figure S29**. Forest plot of standard mean difference Cohen’s *d* of serum HDL concentration in patients with OL versus patients with OC. HDL, high density lipoproteins OL, Oral leukoplakia; OC, oral cancer; CI, credible interval
**Figure S30**. Forest plot of standard mean difference Cohen’s *d* of serum LDL concentration in patients with HC versus patients with OL. LDL, low density lipoproteins; HC, health control; OL, Oral leukoplakia; CI, credible interval
**Figure S31**. Forest plot of standard mean difference Cohen’s *d* of serum LDL concentration in patients with OL versus patients with OC. LDL, low density lipoproteins; OL, Oral leukoplakia; OC, oral cancer; CI, credible interval
**Figure S32**. Forest plot of standard mean difference Cohen’s *d* of serum Albumin concentration in patients with HC versus patients with OL. HC, health control; OL, Oral leukoplakia; CI, credible interval
**Figure S33**. Forest plot of standard mean difference Cohen’s *d* of serum Albumin concentration in patients with OL versus patients with OC. OL, Oral leukoplakia; OC, oral cancer; CI, credible interval
**Figure S34**. Forest plot of standard mean difference Cohen’s *d* of serum Protein concentration in patients with HC versus patients with OL. HC, health control; OL, Oral leukoplakia; CI, credible interval
**Figure S35**. Forest plot of standard mean difference Cohen’s *d* of serum Protein concentration in patients with OL versus patients with OC. OL, Oral leukoplakia; OC, oral cancer; CI, credible interval
**Figure S36**. Forest plot of standard mean difference Cohen’s *d* of serum β2‐M concentration in patients with HC versus patients with OL. β2‐M, β2‐microglobulin; HC, health control; OL, Oral leukoplakia; CI, credible interval
**Figure S37**. Forest plot of standard mean difference Cohen’s *d* of serum β2‐M concentration in patients with OL versus patients with OC. β2‐M, β2‐microglobulin; OL, Oral leukoplakia; OC, oral cancer; CI, credible interval
**Figure S38**. Forest plot of standard mean difference Cohen’s *d* of serum Fucose concentration in patients with HC versus patients with OL. HC, health control; OL, Oral leukoplakia; CI, credible interval
**Figure S39**. Forest plot of standard mean difference Cohen’s *d* of serum Fucose concentration in patients with OL versus patients with OC. OL, Oral leukoplakia; OC, oral cancer; CI, credible interval

## Data Availability

All data generated or analysed during this study are included in this published article and its supplementary information files.
