# Supplementary material for: Potential noninvasive biomarkers for the malignant transformation of oral leukoplakia: A systematic review and meta‐analysis
Source: Cancer Med. 2023 May 18;12(13):14718–30. doi: 10.1002/cam4.6095 (PMC10358244; doi:10.1002/cam4.6095)
Supplement: Supplementary file 1 — Figure S1. Forest plot of standard mean difference Cohen’s d of salivary IL‐6 concentration in patients with HC versus patients with OL. IL‐6, interleukin‐6; HC, health control; OL, Oral leukoplakia; CI, credible interval Figure S2. Forest plot of standard mean difference Cohen’s d of salivary IL‐6 concentration in patients with OL versus patients with OC. IL‐6, interleukin‐6; OL, Oral leukoplakia; OC, oral cancer; CI, credible interval Figure S3. Forest plot of standard mean difference Cohen’s d of salivary TNF‐α concentration in patients with HC versus patients with OL. TNF‐α, tumor necrosis factor alpha; HC, health control; OL, Oral leukoplakia; CI, credible interval Figure S4. Forest plot of standard mean difference Cohen’s d of salivary TNF‐α concentration in patients with OL versus patients with OC. TNF‐α, tumor necrosis factor alpha; OL, Oral leukoplakia; OC, oral cancer; CI, credible interval Figure S5. Forest plot of standard mean difference Cohen’s d of salivary IL‐1α concentration in patients with HC versus patients with OL. IL‐1α, interleukin‐1alpha; HC, health control; OL, Oral leukoplakia; CI, credible interval Figure S6. Forest plot of standard mean difference Cohen’s d of salivary IL‐1α concentration in patients with OL versus patients with OC. IL‐1α, interleukin‐1alpha; OL, Oral leukoplakia; OC, oral cancer; CI, credible interval Figure S7. Forest plot of standard mean difference Cohen’s d of salivary IL‐8 concentration in patients with HC versus patients with OL. IL‐8, interleukin‐8; HC, health control; OL, Oral leukoplakia; CI, credible interval Figure S8. Forest plot of standard mean difference Cohen’s d of salivary IL‐8 concentration in patients with OL versus patients with OC. IL‐8, interleukin‐8; OL, Oral leukoplakia; OC, oral cancer; CI, credible interval Figure S9. Forest plot of standard mean difference Cohen’s d of salivary Copper concentration in patients with HC versus patients with OL. HC, health control; OL, Oral leukoplakia; CI, credi [file CAM4-12-14718-s001.docx]

**Supplementary Material**

**Supplementary Figure S1**. Forest plot of standard mean difference Cohen’s *d* of salivary IL-6 concentration in patients with HC versus patients with OL. IL-6, interleukin-6; HC, health control; OL, Oral leukoplakia; CI, credible interval

**Supplementary Figure S2**. Forest plot of standard mean difference Cohen’s *d* of salivary IL-6 concentration in patients with OL versus patients with OC. IL-6, interleukin-6; OL, Oral leukoplakia; OC, oral cancer; CI, credible interval

**Supplementary Figure S3**. Forest plot of standard mean difference Cohen’s *d* of salivary TNF-α concentration in patients with HC versus patients with OL. TNF-α, tumor necrosis factor alpha; HC, health control; OL, Oral leukoplakia; CI, credible interval

**Supplementary Figure S4**. Forest plot of standard mean difference Cohen’s *d* of salivary TNF-α concentration in patients with OL versus patients with OC. TNF-α, tumor necrosis factor alpha; OL, Oral leukoplakia; OC, oral cancer; CI, credible interval

**Supplementary Figure S5**. Forest plot of standard mean difference Cohen’s *d* of salivary IL-1α concentration in patients with HC versus patients with OL. IL-1α, interleukin-1alpha; HC, health control; OL, Oral leukoplakia; CI, credible interval

**Supplementary Figure S6**. Forest plot of standard mean difference Cohen’s *d* of salivary IL-1α concentration in patients with OL versus patients with OC. IL-1α, interleukin-1alpha; OL, Oral leukoplakia; OC, oral cancer; CI, credible interval

**Supplementary Figure S7**. Forest plot of standard mean difference Cohen’s *d* of salivary IL-8 concentration in patients with HC versus patients with OL. IL-8, interleukin-8; HC, health control; OL, Oral leukoplakia; CI, credible interval

**Supplementary Figure S8**. Forest plot of standard mean difference Cohen’s *d* of salivary IL-8 concentration in patients with OL versus patients with OC. IL-8, interleukin-8; OL, Oral leukoplakia; OC, oral cancer; CI, credible interval

**Supplementary Figure S9**. Forest plot of standard mean difference Cohen’s *d* of salivary Copper concentration in patients with HC versus patients with OL. HC, health control; OL, Oral leukoplakia; CI, credible interval

**Supplementary Figure S10**. Forest plot of standard mean difference Cohen’s *d* of salivary Copper concentration in patients with OL versus patients with OC. OL, Oral leukoplakia; OC, oral cancer; CI, credible interval

**Supplementary Figure S11**. Forest plot of standard mean difference Cohen’s *d* of salivary Zinc concentration in patients with HC versus patients with OL. HC, health control; OL, Oral leukoplakia; CI, credible interval

**Supplementary Figure S12**. Forest plot of standard mean difference Cohen’s *d* of salivary Zinc concentration in patients with OL versus patients with OC. OL, Oral leukoplakia; OC, oral cancer; CI, credible interval

**Supplementary Figure S13**. Forest plot of standard mean difference Cohen’s *d* of salivary LDH concentration in patients with HC versus patients with OL. LDH, lactate dehydrogenase; HC, health control; OL, Oral leukoplakia; CI, credible interval

**Supplementary Figure S14**. Forest plot of standard mean difference Cohen’s *d* of salivary LDH concentration in patients with OL versus patients with OC. LDH, lactate dehydrogenase; OL, Oral leukoplakia; OC, oral cancer; CI, credible interval

**Supplementary Figure S15**. Forest plot of standard mean difference Cohen’s *d* of serum LSA concentration in patients with HC versus patients with OL. LSA, lipid bound sialic acid; HC, health control; OL, Oral leukoplakia; CI, credible interval

**Supplementary Figure S16**. Forest plot of standard mean difference Cohen’s *d* of serum LSA concentration in patients with OL versus patients with OC. LSA, lipid bound sialic acid; OL, Oral leukoplakia; OC, oral cancer; CI, credible interval

**Supplementary Figure S17**. Forest plot of standard mean difference Cohen’s *d* of serum TSA concentration in patients with HC versus patients with OL. TSA, total sialic acid; HC, health control; OL, Oral leukoplakia; CI, credible interval

**Supplementary Figure S18**. Forest plot of standard mean difference Cohen’s *d* of serum TSA concentration in patients with OL versus patients with OC. TSA, total sialic acid; OL, Oral leukoplakia; OC, oral cancer; CI, credible interval

**Supplementary Figure S19**. Forest plot of standard mean difference Cohen’s *d* of serum IL-6 concentration in patients with HC versus patients with OL. IL-6, interleukin-6; HC, health control; OL, Oral leukoplakia; CI, credible interval

**Supplementary Figure S20**. Forest plot of standard mean difference Cohen’s *d* of serum IL-6 concentration in patients with OL versus patients with OC. IL-6, interleukin-6; OL, Oral leukoplakia; OC, oral cancer; CI, credible interval

**Supplementary Figure S21**. Forest plot of standard mean difference Cohen’s *d* of serum TNF-α concentration in patients with HC versus patients with OL. TNF-α, tumor necrosis factor alpha; HC, health control; OL, Oral leukoplakia; CI, credible interval

**Supplementary Figure S22**. Forest plot of standard mean difference Cohen’s *d* of serum CRP concentration in patients with HC versus patients with OL. CRP, C-reactive protein; HC, health control; OL, Oral leukoplakia; CI, credible interval

**Supplementary Figure S23**. Forest plot of standard mean difference Cohen’s *d* of serum CRP concentration in patients with OL versus patients with OC. CRP, C-reactive protein; OL, Oral leukoplakia; OC, oral cancer; CI, credible interval

**Supplementary Figure S24**. Forest plot of standard mean difference Cohen’s *d* of serum TC concentration in patients with HC versus patients with OL. TC, total cholesterol; HC, health control; OL, Oral leukoplakia; CI, credible interval

**Supplementary Figure S25**. Forest plot of standard mean difference Cohen’s *d* of serum TC concentration in patients with OL versus patients with OC. TC, total cholesterol; OL, Oral leukoplakia; OC, oral cancer; CI, credible interval

**Supplementary Figure S26**. Forest plot of standard mean difference Cohen’s *d* of serum TG concentration in patients with HC versus patients with OL. TG, triglycerides; HC, health control; OL, Oral leukoplakia; CI, credible interval

**Supplementary Figure S27**. Forest plot of standard mean difference Cohen’s *d* of serum TG concentration in patients with OL versus patients with OC. TG, triglycerides; OL, Oral leukoplakia; OC, oral cancer; CI, credible interval

**Supplementary Figure S28**. Forest plot of standard mean difference Cohen’s *d* of serum HDL concentration in patients with HC versus patients with OL. HDL, high density lipoproteins HC, health control; OL, Oral leukoplakia; CI, credible interval

**Supplementary Figure S29**. Forest plot of standard mean difference Cohen’s *d* of serum HDL concentration in patients with OL versus patients with OC. HDL, high density lipoproteins OL, Oral leukoplakia; OC, oral cancer; CI, credible interval

**Supplementary Figure S30**. Forest plot of standard mean difference Cohen’s *d* of serum LDL concentration in patients with HC versus patients with OL. LDL, low density lipoproteins; HC, health control; OL, Oral leukoplakia; CI, credible interval

**Supplementary Figure S31**. Forest plot of standard mean difference Cohen’s *d* of serum LDL concentration in patients with OL versus patients with OC. LDL, low density lipoproteins; OL, Oral leukoplakia; OC, oral cancer; CI, credible interval

**Supplementary Figure S32**. Forest plot of standard mean difference Cohen’s *d* of serum Albumin concentration in patients with HC versus patients with OL. HC, health control; OL, Oral leukoplakia; CI, credible interval

**Supplementary Figure S33**. Forest plot of standard mean difference Cohen’s *d* of serum Albumin concentration in patients with OL versus patients with OC. OL, Oral leukoplakia; OC, oral cancer; CI, credible interval

**Supplementary Figure S34**. Forest plot of standard mean difference Cohen’s *d* of serum Protein concentration in patients with HC versus patients with OL. HC, health control; OL, Oral leukoplakia; CI, credible interval

**Supplementary Figure S35**. Forest plot of standard mean difference Cohen’s *d* of serum Protein concentration in patients with OL versus patients with OC. OL, Oral leukoplakia; OC, oral cancer; CI, credible interval

**Supplementary Figure S36**. Forest plot of standard mean difference Cohen’s *d* of serum β2-M concentration in patients with HC versus patients with OL. β2-M, β2-microglobulin; HC, health control; OL, Oral leukoplakia; CI, credible interval

**Supplementary Figure S37**. Forest plot of standard mean difference Cohen’s *d* of serum β2-M concentration in patients with OL versus patients with OC. β2-M, β2-microglobulin; OL, Oral leukoplakia; OC, oral cancer; CI, credible interval

**Supplementary Figure S38**. Forest plot of standard mean difference Cohen’s *d* of serum Fucose concentration in patients with HC versus patients with OL. HC, health control; OL, Oral leukoplakia; CI, credible interval

**Supplementary Figure S39**. Forest plot of standard mean difference Cohen’s *d* of serum Fucose concentration in patients with OL versus patients with OC. OL, Oral leukoplakia; OC, oral cancer; CI, credible interval

**Supplementary Figure S1**. Forest plot of standard mean difference Cohen’s *d* of salivary IL-6 concentration in patients with HC versus patients with OL. IL-6, interleukin-6; HC, health control; OL, Oral leukoplakia; CI, credible interval

**Supplementary Figure S2**. Forest plot of standard mean difference Cohen’s *d* of salivary IL-6 concentration in patients with OL versus patients with OC. IL-6, interleukin-6; OL, Oral leukoplakia; OC, oral cancer; CI, credible interval

**Supplementary Figure S3**. Forest plot of standard mean difference Cohen’s *d* of salivary TNF-α concentration in patients with HC versus patients with OL. TNF-α, tumor necrosis factor alpha; HC, health control; OL, Oral leukoplakia; CI, credible interval

**Supplementary Figure S4**. Forest plot of standard mean difference Cohen’s *d* of salivary TNF-α concentration in patients with OL versus patients with OC. TNF-α, tumor necrosis factor alpha; OL, Oral leukoplakia; OC, oral cancer; CI, credible interval

**Supplementary Figure S5**. Forest plot of standard mean difference Cohen’s *d* of salivary IL-1α concentration in patients with HC versus patients with OL. IL-1α, interleukin-1alpha; HC, health control; OL, Oral leukoplakia; CI, credible interval

**Supplementary Figure S6**. Forest plot of standard mean difference Cohen’s *d* of salivary IL-1α concentration in patients with OL versus patients with OC. IL-1α, interleukin-1alpha; OL, Oral leukoplakia; OC, oral cancer; CI, credible interval

**Supplementary Figure S7**. Forest plot of standard mean difference Cohen’s *d* of salivary IL-8 concentration in patients with HC versus patients with OL. IL-8, interleukin-8; HC, health control; OL, Oral leukoplakia; CI, credible interval

**Supplementary Figure S8**. Forest plot of standard mean difference Cohen’s *d* of salivary IL-8 concentration in patients with OL versus patients with OC. IL-8, interleukin-8; OL, Oral leukoplakia; OC, oral cancer; CI, credible interval

**Supplementary Figure S9**. Forest plot of standard mean difference Cohen’s *d* of salivary Copper concentration in patients with HC versus patients with OL. HC, health control; OL, Oral leukoplakia; CI, credible interval

**Supplementary Figure S10**. Forest plot of standard mean difference Cohen’s *d* of salivary Copper concentration in patients with OL versus patients with OC. OL, Oral leukoplakia; OC, oral cancer; CI, credible interval

**Supplementary Figure S11**. Forest plot of standard mean difference Cohen’s *d* of salivary Zinc concentration in patients with HC versus patients with OL. HC, health control; OL, Oral leukoplakia; CI, credible interval

**Supplementary Figure S12**. Forest plot of standard mean difference Cohen’s *d* of salivary Zinc concentration in patients with OL versus patients with OC. OL, Oral leukoplakia; OC, oral cancer; CI, credible interval

**Supplementary Figure S13**. Forest plot of standard mean difference Cohen’s *d* of salivary LDH concentration in patients with HC versus patients with OL. LDH, lactate dehydrogenase; HC, health control; OL, Oral leukoplakia; CI, credible interval

**Supplementary Figure S14**. Forest plot of standard mean difference Cohen’s *d* of salivary LDH concentration in patients with OL versus patients with OC. LDH, lactate dehydrogenase; OL, Oral leukoplakia; OC, oral cancer; CI, credible interval

**Supplementary Figure S15**. Forest plot of standard mean difference Cohen’s *d* of serum LSA concentration in patients with HC versus patients with OL. LSA, lipid bound sialic acid; HC, health control; OL, Oral leukoplakia; CI, credible interval

**Supplementary Figure S16**. Forest plot of standard mean difference Cohen’s *d* of serum LSA concentration in patients with OL versus patients with OC. LSA, lipid bound sialic acid; OL, Oral leukoplakia; OC, oral cancer; CI, credible interval

**Supplementary Figure S17**. Forest plot of standard mean difference Cohen’s *d* of serum TSA concentration in patients with HC versus patients with OL. TSA, total sialic acid; HC, health control; OL, Oral leukoplakia; CI, credible interval

**Supplementary Figure S18**. Forest plot of standard mean difference Cohen’s *d* of serum TSA concentration in patients with OL versus patients with OC. TSA, total sialic acid; OL, Oral leukoplakia; OC, oral cancer; CI, credible interval

**Supplementary Figure S19**. Forest plot of standard mean difference Cohen’s *d* of serum IL-6 concentration in patients with HC versus patients with OL. IL-6, interleukin-6; HC, health control; OL, Oral leukoplakia; CI, credible interval

**Supplementary Figure S20**. Forest plot of standard mean difference Cohen’s *d* of serum IL-6 concentration in patients with OL versus patients with OC. IL-6, interleukin-6; OL, Oral leukoplakia; OC, oral cancer; CI, credible interval

**Supplementary Figure S21**. Forest plot of standard mean difference Cohen’s *d* of serum TNF-α concentration in patients with HC versus patients with OL. TNF-α, tumor necrosis factor alpha; HC, health control; OL, Oral leukoplakia; CI, credible interval

**Supplementary Figure S22**. Forest plot of standard mean difference Cohen’s *d* of serum CRP concentration in patients with HC versus patients with OL. CRP, C-reactive protein; HC, health control; OL, Oral leukoplakia; CI, credible interval

**Supplementary Figure S23**. Forest plot of standard mean difference Cohen’s *d* of serum CRP concentration in patients with OL versus patients with OC. CRP, C-reactive protein; OL, Oral leukoplakia; OC, oral cancer; CI, credible interval

**Supplementary Figure S24**. Forest plot of standard mean difference Cohen’s *d* of serum TC concentration in patients with HC versus patients with OL. TC, total cholesterol; HC, health control; OL, Oral leukoplakia; CI, credible interval

**Supplementary Figure S25**. Forest plot of standard mean difference Cohen’s *d* of serum TC concentration in patients with OL versus patients with OC. TC, total cholesterol; OL, Oral leukoplakia; OC, oral cancer; CI, credible interval

**Supplementary Figure S26**. Forest plot of standard mean difference Cohen’s *d* of serum TG concentration in patients with HC versus patients with OL. TG, triglycerides; HC, health control; OL, Oral leukoplakia; CI, credible interval

**Supplementary Figure S27**. Forest plot of standard mean difference Cohen’s *d* of serum TG concentration in patients with OL versus patients with OC. TG, triglycerides; OL, Oral leukoplakia; OC, oral cancer; CI, credible interval

**Supplementary Figure S28**. Forest plot of standard mean difference Cohen’s *d* of serum HDL concentration in patients with HC versus patients with OL. HDL, high density lipoproteins HC, health control; OL, Oral leukoplakia; CI, credible interval

**Supplementary Figure S29**. Forest plot of standard mean difference Cohen’s *d* of serum HDL concentration in patients with OL versus patients with OC. HDL, high density lipoproteins OL, Oral leukoplakia; OC, oral cancer; CI, credible interval

**Supplementary Figure S30**. Forest plot of standard mean difference Cohen’s *d* of serum LDL concentration in patients with HC versus patients with OL. LDL, low density lipoproteins; HC, health control; OL, Oral leukoplakia; CI, credible interval

**Supplementary Figure S31**. Forest plot of standard mean difference Cohen’s *d* of serum LDL concentration in patients with OL versus patients with OC. LDL, low density lipoproteins; OL, Oral leukoplakia; OC, oral cancer; CI, credible interval

**Supplementary Figure S32**. Forest plot of standard mean difference Cohen’s *d* of serum Albumin concentration in patients with HC versus patients with OL. HC, health control; OL, Oral leukoplakia; CI, credible interval

**Supplementary Figure S33**. Forest plot of standard mean difference Cohen’s *d* of serum Albumin concentration in patients with OL versus patients with OC. OL, Oral leukoplakia; OC, oral cancer; CI, credible interval

**Supplementary Figure S34**. Forest plot of standard mean difference Cohen’s *d* of serum Protein concentration in patients with HC versus patients with OL. HC, health control; OL, Oral leukoplakia; CI, credible interval

**Supplementary Figure S35**. Forest plot of standard mean difference Cohen’s *d* of serum Protein concentration in patients with OL versus patients with OC. OL, Oral leukoplakia; OC, oral cancer; CI, credible interval

**Supplementary Figure S36**. Forest plot of standard mean difference Cohen’s *d* of serum β2-M concentration in patients with HC versus patients with OL. β2-M, β2-microglobulin; HC, health control; OL, Oral leukoplakia; CI, credible interval

**Supplementary Figure S37**. Forest plot of standard mean difference Cohen’s *d* of serum β2-M concentration in patients with OL versus patients with OC. β2-M, β2-microglobulin; OL, Oral leukoplakia; OC, oral cancer; CI, credible interval

**Supplementary Figure S38**. Forest plot of standard mean difference Cohen’s *d* of serum Fucose concentration in patients with HC versus patients with OL. HC, health control; OL, Oral leukoplakia; CI, credible interval

**Supplementary Figure S39**. Forest plot of standard mean difference Cohen’s *d* of serum Fucose concentration in patients with OL versus patients with OC. OL, Oral leukoplakia; OC, oral cancer; CI, credible interval
